# Clinical and Molecular Epidemiology of Staphylococcus argenteus Infections in Thailand

**DOI:** 10.1128/JCM.03049-14

**Published:** 2015-02-19

**Authors:** Janjira Thaipadungpanit, Premjit Amornchai, Emma K. Nickerson, Gumphol Wongsuvan, Vanaporn Wuthiekanun, Direk Limmathurotsakul, Sharon J. Peacock

**Affiliations:** a Mahidol-Oxford Tropical Medicine Research Unit, Faculty of Tropical Medicine, Mahidol University, Bangkok, Thailand; b Cambridge University Hospitals NHS Foundation Trust, Cambridge, United Kingdom; c Department of Tropical Medicine and Hygiene, Faculty of Tropical Medicine, Mahidol University, Bangkok, Thailand; d Department of Microbiology and Immunology, Faculty of Tropical Medicine, Mahidol University, Bangkok, Thailand; e Department of Medicine, University of Cambridge, Addenbrooke's Hospital, Cambridge, United Kingdom

## Abstract

Molecular typing of 246 Staphylococcus aureus isolates from unselected patients in Thailand showed that 10 (4.1%) were actually Staphylococcus argenteus. Contrary to the suggestion that S. argenteus is less virulent than S. aureus, we demonstrated comparable rates of morbidity, death, and health care-associated infection in patients infected with either of these two species.

## TEXT

The population genetic structure of Staphylococcus aureus has been extensively described using multilocus sequence typing (MLST) (1). The original primers failed to amplify all 7 MLST loci for a small proportion of presumptive S. aureus isolates, resulting in the delayed description of an early branching lineage that was initially referred to as clonal complex 75 (CC75). Sequence type 1223 (ST1223), which belongs to the same lineage but does not cluster within CC75, was subsequently described (2, 3). The new species name Staphylococcus argenteus was proposed for CC75 and related STs (4), followed by formal taxonomic classification as S. argenteus sp. nov. (5).

Using a single-nucleotide polymorphism (SNP) genotyping method based on MLST, staphylococci belonging to CC75 were found to account for 71% of community-associated methicillin-resistant S. aureus (MRSA) skin and soft tissue infections in indigenous communities in the Northern Territory of Australia (6). Further isolation of CC75 from these communities (3) was followed by reports of its isolation in Cambodia (2), Indonesia (2), Fiji (7), and Trinidad and Tobago (8) and from members of an isolated village in the Amazonian forest in French Guinea (9). Information contained in the MLST database suggests that S. argenteus is also present in Europe and Africa. Overall, this indicates widespread dissemination of S. argenteus, and a report of its isolation from the feces of the straw-colored fruit bat in Nigeria (10) indicates that it may be carried by other animal species.

Isolation of S. argenteus in Thailand has not been reported, but based on isolation elsewhere in Southeast Asia we presumed that it was present. This is made more plausible by the fact that S. argenteus cannot be distinguished from S. aureus using routine diagnostic microbiology identification (2, 6, 7, 9). We investigated the presence of S. argenteus in Thailand through a reevaluation of S. aureus isolates obtained during a prospective study of 270 consecutive patients presenting with invasive infection at Sappasithiprasong Hospital in Northeast Thailand between November 2006 and November 2007 (11). All the isolates were originally identified based on Gram staining and the coagulase test and further evaluated for methicillin susceptibility and the presence of *pvl* and *mecA* and stored at −80°C (11).

Of the 270 isolates originally stored, 246 (91.1%) were available for genotyping using a combination of PCR and multilocus sequence typing (MLST). Of these isolates, 37 were MRSA. As ST239 is the predominant MRSA lineage in Thailand (12), MRSA isolates were initially screened using an ST239-specific PCR assay (12). All non-ST239 isolates were then typed using MLST as described previously (1), except for modifications in primer design to improve PCR amplification (see Table S1 in the supplemental material). Allele and ST assignments were performed using the MLST database (http://saureus.mlst.net). S. argenteus isolates in the study collection were identified based on phylogenetic clustering. To enrich the population for relevant genotypes, 38 STs were downloaded from the MLST database (http://www.mlst.net) as a reference collection. These were selected following an initial analysis in which phylogenetic trees were drawn for each S. aureus MLST locus using all of the data held in the MLST database (not shown). This demonstrated that several *arcC* alleles (36, 151, 207, and 271) and *pta* alleles (39, 107, 145, 175, 198, 256, 268, and 287) clustered in a single branch containing known S. argenteus isolates and were not shared with S. aureus (see Fig. S1 and S2 in the supplemental material). The remaining five alleles were shared between the two species. Based on this, we selected all STs that contained these particular *arcC* and *pta* alleles. The 38 STs were visualized using eBURST with a cutoff of 4 or more shared loci, which demonstrated that 33 STs belonged to one of four CCs: CC75 (*n =* 4 STs), CC2198 (*n =* 8 STs), CC2483 (*n =* 10 STs), or CC1594 (*n =* 11 STs). The remaining 5 STs were either singletons (ST2225, ST2351, or ST2470) or paired (ST2596 and ST2793). A maximum-likelihood tree was reconstructed from concatenated sequences of 7 MLST loci using PhyML version 3.0.1 (13). The CLC Main Workbench version 7.0 (Qiagen, USA) was used to edit and display the tree.

**FIG 1:**
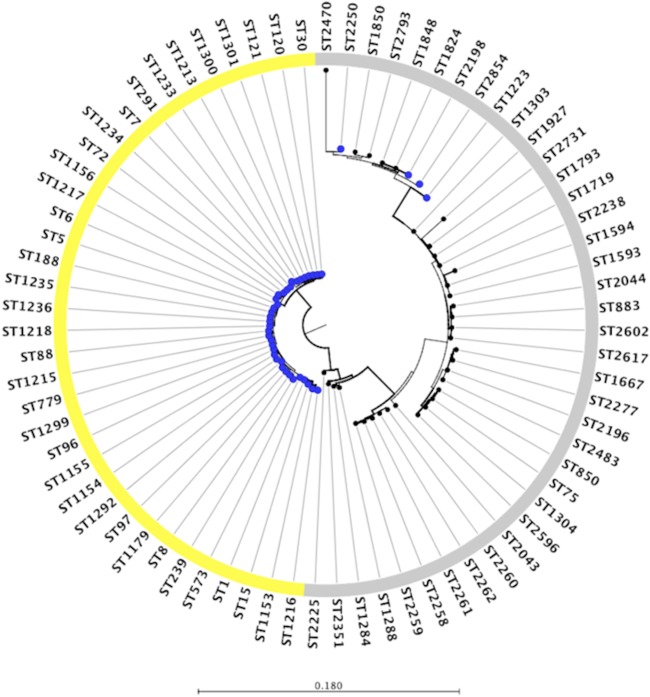
Phylogenetic tree of 246 putative S. aureus isolates based on concatenated sequences of 7 MLST loci using the maximum-likelihood method. Blue dots represent STs identified for the 246 study isolates, and black dots represent 38 STs downloaded from the MLST website (http://www.mlst.net). The colored ring denotes known or proposed species for each ST (yellow, S. aureus; gray, S. argenteus).

A total of 40 STs were identified in the 246 Thai isolates. The predominant genotype in the collection was ST121 (95/246, 38.6%); the frequency of STs for the collection is summarized in Fig. S3 in the supplemental material. A maximum-likelihood phylogenetic tree was reconstructed based on concatenated sequences of 7 MLST loci for these STs, together with the 38 reference S. argenteus STs from the MLST website (Fig. 1). The phylogenetic tree divided the Thai collection into two major branches containing 36 STs that were S. aureus and 4 STs that clustered with the S. argenteus reference sequences. Three groups were observed for all of the S. argenteus data, one of which contained all four STs (representing 10 isolates) from the Thai collection. Three of these four STs were identified previously (ST1223 reported from Cambodia, Australia, and French Guiana, ST2250 from the United Kingdom, and ST2198 from Australia and Germany) (2, 3, 4, 9). The fourth ST (ST2854) is novel. Based on this phylogenetic analysis, we concluded that 10/246 isolates (4.1%) from an unselected consecutive collection of S. aureus isolates from Thailand should be recategorized as S. argenteus. Our findings confirm that this species is endemic in Thailand and provides additional evidence to suggest that S. argenteus may be more widely distributed across Southeast Asia.

S. argenteus isolates identified in Australia are predominantly resistant to methicillin, but those identified elsewhere are susceptible to methicillin; isolates tested to date are universally *pvl* negative (6–9, 14). All 10 Thai S. argenteus isolates were methicillin susceptible, *mecA* negative, and *pvl* negative. The frequencies of methicillin resistance and *pvl* positivity for the entire Thai collection are summarized in Fig. S3 in the supplemental material.

S. argenteus isolates characteristically lack staphyloxanthin, a carotenoid pigment responsible for the characteristic golden colony color (4). All 10 Thai S. argenteus grew on blood agar as white colonies, which is consistent with this. Staphyloxanthin confers resistance against oxidative stress and neutrophil killing (15), leading to the hypothesis that S. argenteus may be less virulent than S. aureus (4). This is supported by the findings of a study showing that an S. argenteus isolate (ST1850) was associated with lower virulence than S. aureus in murine sepsis and skin infection models (16). Transformation of this isolate with a plasmid carrying the gene encoding staphyloxanthin led to increased resistance to oxidative stress *in vitro* but no change in resistance to neutrophil killing or *in vivo* virulence. A study that defined the frequency of S. argenteus in three different clinical collections in northern Australia reported that this species was predominantly associated with skin and soft tissue infections but rarely with bacteremia (1% [3/220] of methicillin-susceptible S. aureus [MSSA] bacteremias and 0% [0/47] of MRSA bacteremias) (16).

We tested the hypothesis that S. argenteus isolates were not associated with severe disease in our Thai cohort. Clinical details and outcomes from S. argenteus infection are shown in Table 1. Most patients (8/10, 80%) had community-acquired infection, but two had health care-associated infection, indicating that S. argenteus may cause nosocomial infection. We noted that S. argenteus was strongly associated with skin and soft tissue infections or superficial abscesses (8/10 cases), which is consistent with previous reports. However, three patients with S. argenteus infection had bacteremia, and two patients had bone and joint infections, indicating the ability of S. argenteus to cause invasive disease (see Table S2 in the supplemental material). Two patients infected with S. argenteus died, one as a direct result of the infection. In general, disease characteristics and outcomes were not different between the patients infected with S. argenteus and those infected with MSSA. However, the median age of the patients infected with S. argenteus was higher than that of patients infected with MSSA (56.5 versus 38; *P* = 0.04), and patients infected with S. argenteus were more likely to have diabetes and renal disease than patients infected with MSSA (Table 1).

**TABLE 1:** Clinical manifestations and outcomes for patients infected with MSSA or S. argenteus

Clinical parameter	Data for patients with^*a*^:		*P* value^*b*^
	MSSA	S. argenteus	
Total cases	199	10	
Age (yr)	38 (14–59, 1–84)	56.5 (41–66, 11–73)	0.04
Sex (male)	118 (59)	7 (70)	0.74
Blood culture-positive isolates	56 (28)	3 (30)	>0.99
Place of acquisition			
Community acquired	172 (86)	8 (80)	0.63
Health care associated^*c*^	27 (14)	2 (20)	
Underlying medical condition^*d*^	65 (33)	7 (70)	0.03
Cardiac disease^*e*^	12 (6)	1 (10)	0.48
Diabetes mellitus	29 (15)	6 (60)	0.002
Renal disease^*f*^	15 (8)	6 (60)	<0.001
Immunosuppression^*g*^	25 (13)	2 (20)	0.62
Lung disease^*h*^	9 (5)	1 (10)	0.39
Pattern of disease			
Identified site, bacteremia only	17 (9)	2 (20)	0.19
1 identified site	155 (78)	6 (60)	
>1 identified site	27 (13)	2 (20)	
Other identifiable sites of infection			
Superficial abscess	81 (41)	4 (40)	0.15
Deep abscess	45 (23)	0	
Other skin and soft tissue infections	21 (11)	4 (40)	
Bone and joint infections	24 (12)	2 (20)	
Prosthetic material infection	13 (7)	0	
Respiratory infection	14 (7)	0	
Endocarditis	6 (3)	0	
Other infection	7 (4)	0	
Outcome			
Cured	150 (75)	7 (70)	0.58
Treatment failure	11 (6)	1 (10)	
Death due to S. aureus	26 (13)	1 (10)	
Death due to other causes	12 (6)	1 (10)	

a Data shown are number (%) or median (interquartile range, range).

b *P* values were estimated using Fisher's exact test. Values for age were estimated using the Mann-Whitney test.

c Health care-associated infections included nosocomial and nonnosocomial health care-associated infections (12).

d Past medical history of any underlying chronic medical conditions reported by the patients and/or relatives or recorded in the medical notes.

e “Cardiac disease” comprised congenital heart disease, valvular heart disease (including rheumatic heart disease), ischemic disease, or arrhythmias (including heart block requiring pacemaker).

f “Renal disease” included end-stage renal failure with long-term dialysis and chronic renal failure (not on dialysis) due to diabetes mellitus, systematic lupus erythematous, multiple myeloma, glomerulonephritis, or an unknown etiology.

g Immunosuppression included that from HIV, chemotherapy, untreated leukemia, radiotherapy, or immunosuppressive medications, including >30 mg/day of prednisolone for >1 week.

h “Lung disease” comprised previously treated tuberculosis, previous empyema, lung cancer, long-term tracheostomy, chronic obstructive pulmonary disease, or asthma.

A limitation of this work is that the study cohort included only patients with S. aureus isolated from a normally sterile site. This is likely to have led to an underrepresentation of minor skin and soft tissue infections, which may have resulted in an underestimation of the true prevalence of S. argenteus. Our data demonstrate, however, that S. argenteus may be associated with serious morbidity, death, and nosocomial infection, particularly in patients with underlying diseases, such as diabetes mellitus and renal diseases.

## Supplementary Material

Supplemental material
