# Supplementary material for: Clinical and Molecular Epidemiology of Staphylococcus argenteus Infections in Thailand
Source: J Clin Microbiol. 2015 Feb 19;53(3):1005–8. doi: 10.1128/JCM.03049-14 (PMC4390622; doi:10.1128/JCM.03049-14)
Supplement: Supplemental material [file supp_53_3_1005__index.html]

Supplemental material 

# Clinical and Molecular Epidemiology of Staphylococcus argenteus Infections in Thailand

## Supplemental material

**Files in this Data Supplement:**

- Supplemental file 1 -

  Table S1 (Primers and PCR conditions for MLST)

  PDF, 118K
- Supplemental file 2 -

  Table S2 (*S. argenteus* patient characteristics)

  PDF, 85K
- Supplemental file 3 -

  Fig. S1 (Phylogenetic tree of *arcC*)

  PDF, 569K
- Supplemental file 4 -

  Fig. S2 (Phylogenetic tree of *pta*)

  PDF, 577K
- Supplemental file 5 -

  Fig. S3 (Distribution of *pvl* by sequence type and methicillin resistance)

  PDF, 199K
