# Supplementary material for: Clinical and Molecular Epidemiology of Staphylococcus argenteus Infections in Thailand
Source: J Clin Microbiol. 2015 Feb 19;53(3):1005–8. doi: 10.1128/JCM.03049-14 (PMC4390622; doi:10.1128/JCM.03049-14)
Supplement: Supplemental material [file JCM.03049-14_zjm999094096so1.pdf]

Supplementary Table 1. Primers and PCR conditions for MLST

Locus	Annealing temperature and $MgCl_2$[1]	Primer[2]	Primer sequence (5´-3´)
aroE	45°C and 4.5 mM	*aro-me-f	tttccagccatacccgc
		*aro-me-r	atgaaatttgcagttataggaaa
glpF	51°C and 4.5 mM	*glpF*-f	ctaggaactgcaatcttaatcc
		**glpF*-me-r	ccggcaattggtcctaagat
gmk	45°C and 1.5 mM	*gmk*-f	atcgttttatcgggaccatc
		**gmk*-me-r	agtgctcagcttctacaataca
tpi	51°C and 4.5 mM	*tpi-me*-f	cacaggtgaaacgtctccagt
		**tpi-me*-r	tattttgcaccttctaaca
yqiL	45°C and 1.5 mM	**yqiL*-f	attagcagcagcatacaggac
		yqiL-r	cgttgaggaatcgatactggaac

[1]PCR reactions were carried out a total volume of 25 µl containing 1×PCR buffer, $MgCl_2$ as shown in the table, 0.2 mM dNTP (Roche Applied Science, Germany), 5 pmol of each primer, 1.25 unit of Taq DNA polymerase (Roche Applied Science, Germany), and 10-120 ng of bacterial gDNA. PCR conditions were one cycle of 95°C for 2 min, following by 35 cycles of 95°C for 15s, the annealing temperature shown in the table, 15s; 72°C, 30s, and 72°C, 7 min. For *S. argenteus*, increased gDNA templates (two fold) and primer (three fold for *glp* only) was necessary to increase amplification yield.

[2]The primers shown are limited to the five MLST loci for which at least one primer (marked with asterisk) was re-designed during this study. The primers used for the remaining two loci were as described previously (1).

References

1. Enright MC, Day NP, Davies CE, Peacock SJ, Spratt BG. 2000. Multilocus sequence typing for characterization of methicillin-resistant and methicillin-susceptible clones of *Staphylococcus aureus*. J Clin Microbiol **38**:1008-1015.
