# Supplementary material for: Clinical and Molecular Epidemiology of Staphylococcus argenteus Infections in Thailand
Source: J Clin Microbiol. 2015 Feb 19;53(3):1005–8. doi: 10.1128/JCM.03049-14 (PMC4390622; doi:10.1128/JCM.03049-14)
Supplement: Supplemental material [file JCM.03049-14_zjm999094096so2.pdf]

Supplementary Table 2. *S. argenteus* patient characteristics

No.	Age (years)	Sex	ST	Place of acquisition	Blood culture positive	Clinical pattern of disease	Outcome	Underlying disease
1	71	male	1223	community-acquired	no	necrotising fasciitis with skin and soft tissue abscess (scalp)	cured	diabetes mellitus, renal disease and lung disease
2	66	female	1223	community-acquired	no	skin and soft tissue abscess (toe)	cured	none
3	11	female	2198	community-acquired	no	infected skin wound following trauma	cured	none
4	51	male	2250	community-acquired	no	diabetic foot infection	treatment failure	diabetes mellitus and renal disease
5	47	male	2250	community-acquired	no	necrotising fasciitis	cured	diabetes mellitus and immunosuppression
6	41	female	2854	community-acquired	no	skin and soft tissue abscesses (buttock, axilla)	cured	none
7	38	male	1223	community-acquired	yes	osteomyelitis with bacteraemia	cured	diabetes mellitus and renal disease
8	73	male	1223	community-acquired	yes	bacteraemia with no additional foci identified	death due to the infection	diabetes mellitus and renal disease
9	62	male	2250	healthcare-associated	no	infected tophaceous gout wounds	cured	diabetes mellitus and renal disease
10	66	male	1223	healthcare-associated	yes	bacteraemia with no additional foci identified	death from other causes	cardiac disease, renal disease and immunosuppression
