# Supplementary material for: Clinical and Molecular Epidemiology of Staphylococcus argenteus Infections in Thailand
Source: J Clin Microbiol. 2015 Feb 19;53(3):1005–8. doi: 10.1128/JCM.03049-14 (PMC4390622; doi:10.1128/JCM.03049-14)
Supplement: Supplemental material [file JCM.03049-14_zjm999094096so3.pdf]

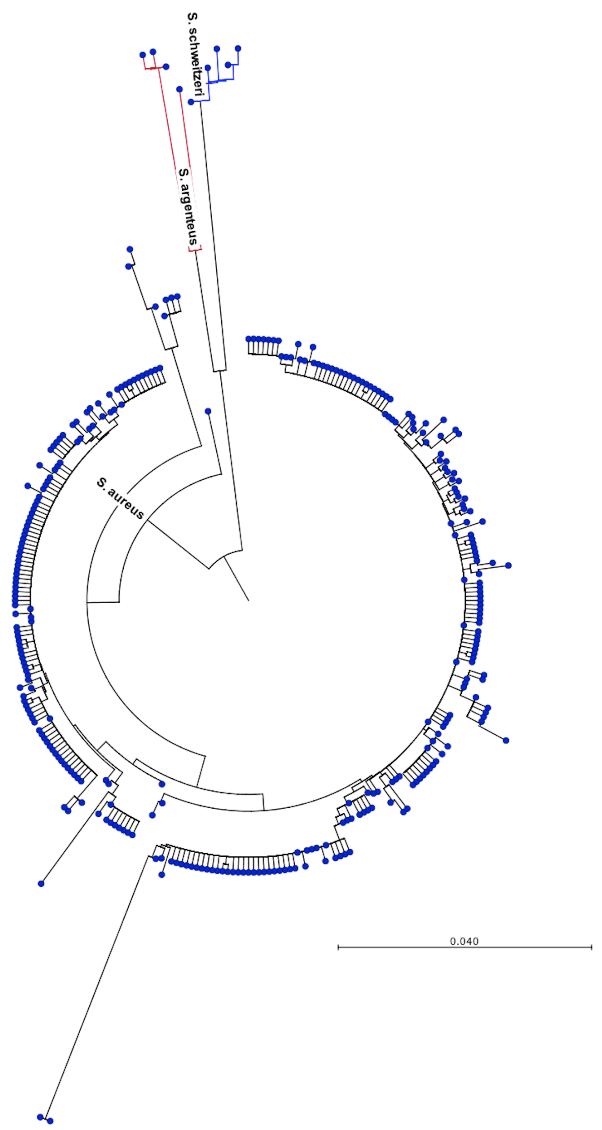

Supplementary Figure 1. Phylogenetic tree of *arcC*

Phylogenetic tree based on all of the available allele sequences for *arcC*, one of the seven *S. aureus* MLST loci. The tree demonstrates two divergence branches, *S. argenteus* and *S. schweitzeri*, that are distinct and genetically distant from *S. aureus*. *S. argenteus* contained four distinct alleles for this locus.
