# Supplementary material for: Clinical and Molecular Epidemiology of Staphylococcus argenteus Infections in Thailand
Source: J Clin Microbiol. 2015 Feb 19;53(3):1005–8. doi: 10.1128/JCM.03049-14 (PMC4390622; doi:10.1128/JCM.03049-14)
Supplement: Supplemental material [file JCM.03049-14_zjm999094096so4.pdf]

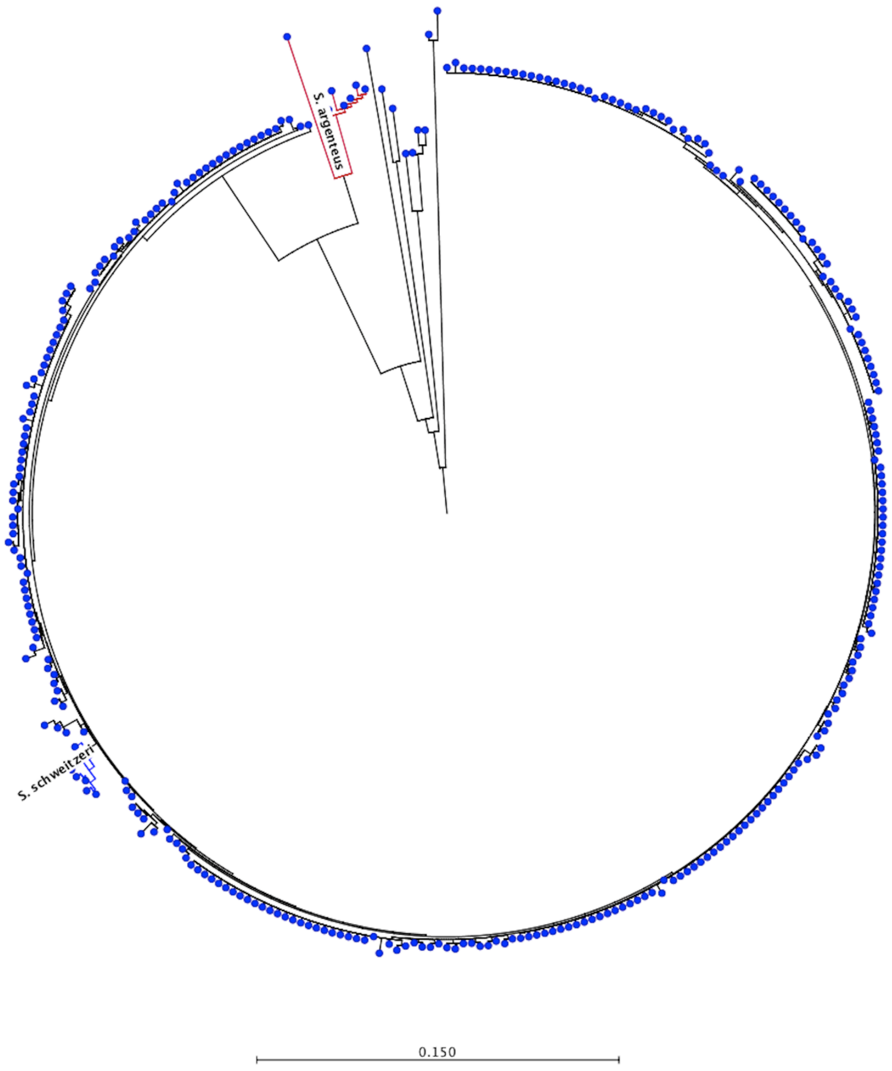

Supplementary Figure 2. Phylogenetic tree of *pta*
Phylogenetic tree based on all of the available allele sequences for *pta*, one of the seven *S. aureus* MLST loci. The tree demonstrates several divergence branches. One of these is a cluster of *S. argenteus* that is distinct and genetically distant from *S. aureus*. Eight alleles associated with known *S. argenteus* isolates are in the cluster. *S. schweitzeri* and *S. aureus* are genetically close related based on *pta* locus.
