# Supplementary figures and images for: Clinical and Molecular Epidemiology of Staphylococcus argenteus Infections in Thailand
Source: J Clin Microbiol. 2015 Feb 19;53(3):1005–8. doi: 10.1128/JCM.03049-14 (PMC4390622; doi:10.1128/JCM.03049-14)

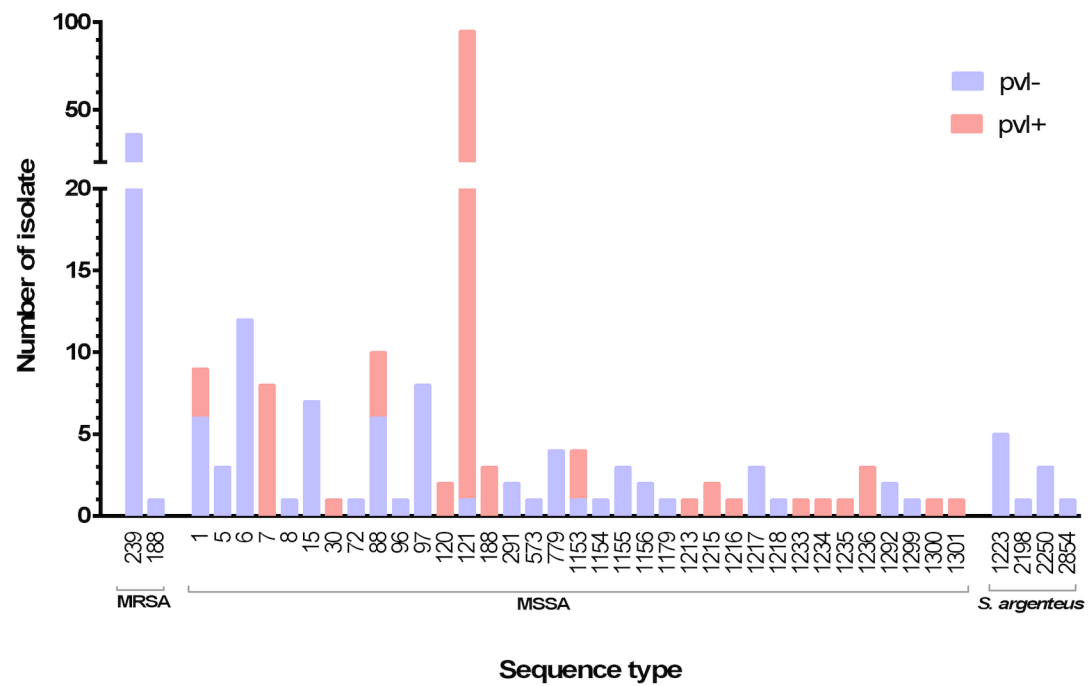

Supplementary Figure 3. Distribution of *pvl* by sequence type and methicillin resistance

Supplement: Supplemental material [file JCM.03049-14_zjm999094096so5.pdf]
